# Predictability of COVID-19-related morbidity and mortality based on model estimations to establish proactive protocols of countermeasures

**DOI:** 10.1038/s41598-021-93932-z

**Published:** 2021-07-15

**Authors:** Göran Svensson, Rocio Rodriguez, Carmen Padin

**Affiliations:** 1 Kristiania University College, Oslo, Norway; 2 University of Murcia, Murcia, Spain; 3 Vigo University, Vigo, Spain

**Keywords:** Health policy, Public health

## Abstract

The COVID-19 pandemic (SARS-CoV-2) has revealed the need for proactive protocols to react and act, imposing preventive and restrictive countermeasures on time in any society. The extent to which confirmed cases can predict the morbidity and mortality in a society remains an unresolved issue. The research objective is therefore to test a generic model’s predictability through time, based on percentage of confirmed cases on hospitalized patients, ICU patients and deceased. This study reports the explanatory and predictive ability of COVID-19-related healthcare data, such as whether there is a spread of a contagious and virulent virus in a society, and if so, whether the morbidity and mortality can be estimated in advance in the population. The model estimations stress the implementation of a pandemic strategy containing a proactive protocol entailing what, when, where, who and how countermeasures should be in place when a virulent virus (e.g. SARS-CoV-1, SARS-CoV-2 and MERS) or pandemic strikes next time. Several lessons for the future can be learnt from the reported model estimations. One lesson is that COVID-19-related morbidity and mortality in a population is indeed predictable. Another lesson is to have a proactive protocol of countermeasures in place.

## Introduction

The COVID-19 pandemic (SARS-CoV-2) has revealed the need for proactive protocols to react and act, imposing preventive and restrictive counter measures on time in a given society. The number of confirmed COVID-19 cases drives the pandemic across societies. The research question is to what extent confirmed COVID-19 cases through time can predict the number of hospitalized and intensive care unit (ICU) COVID-19 patients, as well as the number of deceased by COVID-19.

To the best of the authors knowledge, the predictability of morbidity (i.e. hospitalized patients and ICU patients) and mortality (i.e. deceased) through time based on confirmed cases has received only minor and insufficient attention^1,2^. Consequently, it remains unresolved to what extent confirmed cases can predict the number of hospitalized patients, ICU patients and deceased in a society. The research objective is therefore to test a generic model’s predictability through time, based on the percentage of confirmed cases, on hospitalized patients, ICU patients and deceased. The aim is to establish a logic of model estimation for COVID-19-related morbidity and mortality in the society that may also be applied in other settings of virulent viruses and future pandemics.

**Figure 1:**
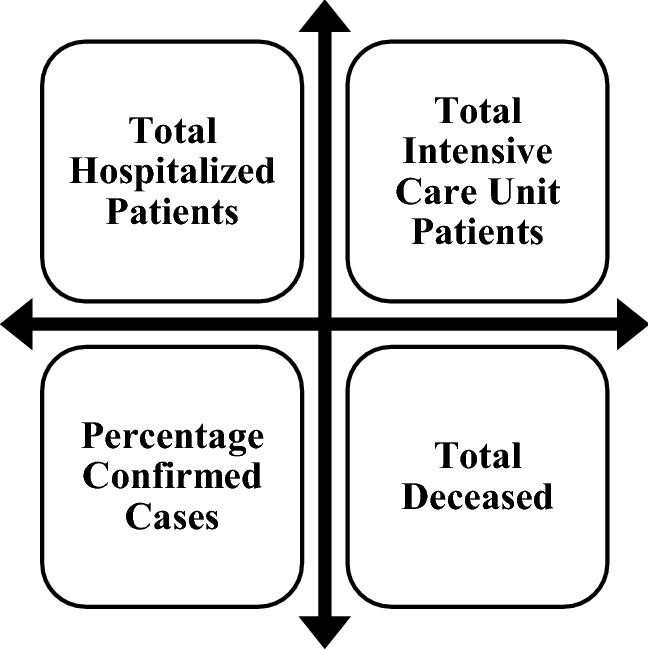
Model of COVID-19 related morbidity and mortality.

The framework of model estimation consists of four COVID-19-related healthcare variables (see Fig. 1) as follows: (1) weekly percentage of confirmed cases; (2) weekly totals of hospitalized patients per day; (3) weekly totals of ICU patients per day; and (4) weekly totals of deceased per day.

The COVID-19-related healthcare data applied in this study has been gathered from the Swedish Agency of Public Health^3^, the Swedish National Board of Health and Welfare, and the Swedish Intensive Care Registry—SIR^4,5^. Consequently, the data used has been obtained directly from the primary source of origin (i.e. not indirectly and from secondary sources) for each variable, and the quality has been assured by each entity. Data validity and reliability has therefore been safeguarded in the data-collection process.

## Results

We test the model estimations of COVID-19-related morbidity and mortality, based on linear and quadratic regression analyses. However, the quadratic analyses are not reported, as no significantly curved distributions were found. Consequently, only a series of linear regression analyses are reported. IBM SPSS Statistics 26.0 software has been used.

The dependent variable was set as the weekly percentage of confirmed cases, while the independent variable was set as either weekly totals of hospitalized patients per day, weekly totals of ICU patients per day or weekly totals of deceased per day, as shown in Table 1. The model estimations also report time-delays from 0 to 4 weeks between the dependent and independent variables. Furthermore, the regression analyses are divided into three time-periods, namely first wave, second wave, and both waves combinéd. The methodology section contains additional details regarding the analyses undertaken to enable replications by others with other sets of healthcare data and pandemic as well as epidemic settings.

**Table 1:** Regression statistics.

Time-period 2020	R	R-square	Adjusted R-square	Standard error of the estimate	Unstandardized beta (predictor)	Coefficient standard error	t	Significance
**(i) Dependent: Number of hospitilized patients per week *(i.e. time-delays from 0 to 4 weeks)* Independent: weekly percentage confirmed cases *(i.e. week zero)***								
Zero week time-delay–time-period 9/3–27/12								
March–August	0.81	0.65	0.64	2597.07	600.77	89.91	6.68	0.00
September–December	0.94	0.89	0.88	2132.59	978.56	93.69	10.45	0.00
March–December	0.85	0.72	0.71	2682.69	739.70	72.84	10.16	0.00
One week time-delay—time-period 16/3–3/1								
March–August	0.91	0.82	0.81	1841.45	669.39	63.75	10.50	0.00
September–December	0.97	0.95	0.94	1581.35	1077.81	69.47	15.52	0.00
March–December	0.89	0.80	0.79	2379.05	815.16	64.60	12.62	0.00
Two weeks time-delay–ime-period 23/3–10/1								
March–August	0.92	0.85	0.85	1674.18	683.74	57.96	11.80	0.00
September–December	0.99	0.96	0.98	861.22	1132.15	37.83	29.92	0.00
March–December	0.89	0.79	0.78	2556.96	839.99	69.43	12.10	0.00
Three weeks time-delay–time-period 30/3–17/1								
March–August	0.87	0.76	0.75	2170.94	661.32	75.16	8.80	0.00
September–December	0.99	0.98	0.97	1074.14	1107.91	47.19	23.48	0.00
March–December	0.83	0.69	0.69	3147.54	812.15	85.47	9.50	0.00
Four weeks time-delay–time-period 6/4–24/1								
March–August	0.80	0.64	0.63	2707.34	617.38	93.73	6.59	0.00
September–December	0.95	0.89	0.89	2112.08	1000.00	92.78	10.78	0.00
March–December	0.75	0.56	0.66	3837.08	739.19	104.19	7.10	0.00
**(ii) Dependent: Number of ICU patients per week (i.e. time-delays from 0 to 4 weeks) independent: weekly percentage confirmed cases (i.e. week zero)**								
Zero week time-delay–time-period 9/3–27/12								
March–August	0.90	0.81	0.80	593.11	201.04	21.04	9.55	0.00
September–December	0.94	0.88	0.88	266.40	121.10	11.70	10.35	0.00
March–December	0.84	0.71	0.70	658.62	171.61	18.84	9.47	0.00
One week time-delay–time-period 16/3–3/1								
March–August	0.93	0.86	0.85	518.84	207.67	17.97	11.56	0.00
September–December	0.96	0.93	0.92	231.15	134.99	10.15	13.29	0.00
March–December	0.90	0.81	0.80	533.36	182.15	14.48	12.58	0.00
Two weeks time-delay–time-period 23/3–10/1								
March–August	0.91	0.83	0.82	576.18	208.79	19.95	10.47	0.00
September–December	0.98	0.96	0.95	193.33	148.07	8.49	17.44	0.00
March–December	0.91	0.82	0.82	510.15	186.82	13.85	13.49	0.00
Three weeks time-delay–time-period 30/3–17/1								
March–August	0.84	0.70	0.69	754.30	196.71	26.11	7.53	0.00
September–December	0.99	0.98	0.98	131.47	153.38	5.78	26.56	0.00
March–December	0.87	0.75	0.75	603.65	180.51	16.39	11.01	0.00
Four weeks time-delay–time-period 6/4–24/1								
March–August	0.75	0.56	0.54	924.01	177.45	31.99	5.54	0.00
September–December	0.98	0.96	0.96	172.28	147.37	7.57	19.47	0.00
March–December	0.80	0.64	0.63	730.18	165.41	19.83	8.34	0.00
**(iii) Dependent: Number of hospitilized AND ICU patients per week (i.e. time-delays from 0 to 4 weeks) independent: weekly percentage confirmed cases (i.e. week zero)**								
Zero week time-delay–time-period 9/3–27/12								
March–August	0.81	0.66	0.65	3338.74	787.38	115.59	6.81	0.00
September–December	0.94	0.89	0.88	2403.29	1098.80	105.57	10.41	0.00
March–December	0.86	0.74	0.74	3099.22	903.58	84.15	10.74	0.00
One week time-delay–time-period 16/3–3/1								
March–August	0.91	0.83	0.82	2359.68	875.78	81.69	10.72	0.00
September–December	0.97	0.94	0.94	1812.45	1211.85	79.62	15.22	0.00
March–December	0.92	0.84	0.84	2514.83	995.86	68.29	14.58	0.00
Two weeks time-delay–time-period 23/3–10/1								
March–August	0.92	0.85	0.85	2180.06	891.53	75.47	11.81	0.00
September–December	0.99	0.98	0.98	1027.00	1278.92	45.11	28.35	0.00
March–December	0.92	0.84	0.83	2632.27	1025.71	71.48	14.35	0.00
Three weeks time-delay–time-period 30/3–17/1								
March–August	0.87	0.76	0.75	2871.75	857.71	99.42	8.63	0.00
September–December	0.99	0.98	0.98	1118.73	1260.62	49.14	25.65	0.00
March–December	0.86	0.74	0.74	3395.40	992.28	92.20	10.76	0.00
Four weeks time-delay–time-period 6/4–24/1								
March–August	0.80	0.63	0.62	3.582.96	795.79	124.04	6.42	0.00
September–December	0.95	0.91	0.90	2245.40	1147.47	98.64	11.63	0.00
March–December	0.78	0.60	0.59	4281.74	905.29	116.26	7.79	0.00
**(iv) Dependent: Number of deceased patients per week (i.e. time-delays from 0 to 4 weeks) independent: weekly percentage confirmed cases (i.e. week zero)**								
Zero week time-delay–time-period 9/3–27/12								
March–August	0.85	0.72	0.71	119.51	32.39	4.14	7.82	0.00
September–December	0.92	0.85	0.84	94.43	37.54	4.24	8.86	0.00
March–December	0.88	0.77	0.76	110.38	34.24	3.00	11.43	0.00
One week time-delay–time-period 16/3–3/1								
March–August	0.90	0.81	0.80	97.91	34.33	3.39	10.13	0.00
September–December	0.96	0.93	0.92	70.63	41.91	3.10	13.51	0.00
March–December	0.92	0.84	0.83	95.10	36.94	2.58	14.31	0.00
Two weeks time-delay–time-period 23/3–10/1								
March–August	0.86	0.74	0.73	115.20	32.98	3.99	8.27	0.00
September–December	0.98	0.97	0.96	50.33	43.84	2.21	19.83	0.00
March–December	0.89	0.78	0.78	112.33	36.66	3.05	12.02	0.00
Three weeks time-delay–time-period 30/3–17/1								
March–August	0.75	0.56	0.54	153.17	29.07	5.30	5.48	0.00
September–December	0.98	0.97	0.97	47.39	43.35	2.08	20.82	0.00
March–December	0.80	0.64	0.63	148.15	33.88	4.02	8.42	0.00
Four weeks time-delay–time-period 6/4–24/1								
March–August	0.65	0.42	0.40	173.19	25.08	6.00	4.18	0.00
September–December	0.95	0.90	0.89	81.98	39.75	3.60	11.04	0.00
March–December	0.70	0.49	0.48	177.11	29.84	4.81	6.20	0.00

Part (i) of Table 1 reports the regression statistics for the relationship between the weekly percentage of confirmed cases and the weekly totals of hospitalized patients. This shows that the R-square estimates (i.e. R-square and adjusted R-square) range between 0.81 and 0.98 across the three time-periods, but with the R-squares, for the full timeframe for March–December at 0.79–0.80. The model predictors (i.e. unstandardized beta) range between 669.4 and 1132.2, with the model estimation based on the complete timeframe at 815.2–840.0. The model predictors are all significant at 0.00. The COVID-19-related healthcare data confirms a significant relationship between the percentage confirmed cases and hospitalized patients through time.

Part (ii) of Table 1 reports the regression statistics for the relationship between the percentage of confirmed cases and the weekly totals of ICU patients. It shows that the R-square estimates range between 0.69 and 0.97 across the three time-periods, but for March–December at 0.78. The model predictors range between 135.0 and 208.8, with the model estimation based on the complete time-period at 181.2–186.2. The model predictors are all significant at 0.00. Accordingly, the COVID-19-related healthcare data confirms a significant relationship between the percentage of confirmed cases and ICU patients through time.

Furthermore, part (iii) of Table 1 reports the regression statistics for the relationship between the percentage of confirmed cases and those for the weekly totals of hospitalized, including the weekly totals of ICU patients. The table shows that the R-square estimates range between 0.82 and 0.98 across the three time-periods, but for March–December at 0.83–0.84. The model predictors range between 875.8 and 1278.9, with the model estimation based on the complete time-period at 891.3–995.9. The model predictors are all significant at 0.00. Accordingly, the COVID-19-related healthcare data confirms a significant relationship between the percentage of confirmed cases and hospitalized including ICU patients, through time.

Finally, part (iv) of Table 1 reports the regression statistics for the relationship between the percentage of confirmed cases and the weekly totals of deceased patients by COVID-19. It shows that the R-square estimates range between 0.73 and 0.97 across the three time-periods, but with March–December at 0.78–0.84. The model predictors range between 33.0 and 43.8, with the model estimation based on the complete time-period at 36.7–36.9. The estimates are all significant at 0.00. Again, the COVID-19-related healthcare data confirms a significant relationship between the percentage of confirmed cases and deceased through time.

It should also be noted that the explanatory and predictive power of the regression analyses reported between the percentage of confirmed cases on the one hand, and the weekly totals of hospitalized patients, ICU patients and deceased on the other, is consistently higher in the second wave, with R-squares ranging between 0.92 and 0.98, while those of the first wave range between 0.73 and 0.86. This is in line with the recent results reported^6^.

**Figure 2:**
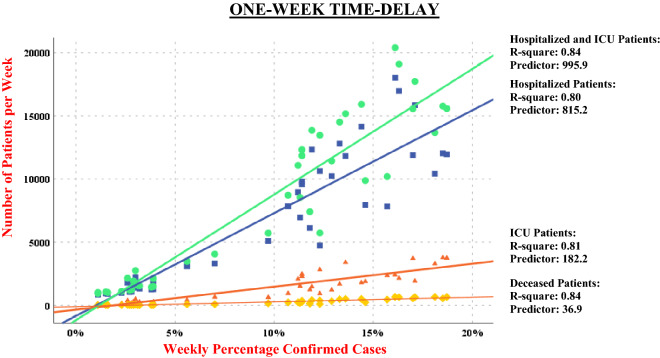
Linear distributions from March to December 2020 with 1 week time-delays.

**Figure 3:**
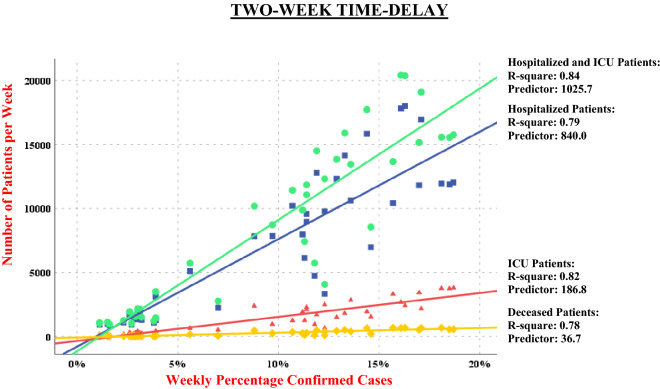
Linear distributions from March to December 2020 with 2-week time-delay.

Figures 2 and 3 displays the March–December regression analyses reported in Table 1. The quadratic regressions and related curved lines as a complement to the linear distributions have also been assessed, but no significant improvement was found (i.e. quadratic lines were only non-significantly curved) and as previously mentioned, therefore not reported.

Each regression line of distribution in the diagram represents one of the four pairs of COVID-19-related healthcare data (i.e. with the same specified time-delay of 1 and 2 weeks): (1) percentage of confirmed cases and weekly totals of hospitalized patients; (2) percentage of confirmed cases and weekly totals of ICU patients; (3) percentage of confirmed cases and weekly totals of hospitalized plus weekly totals of ICU patients; and (4) percentage of confirmed cases and weekly totals of deceased.

Consequently, Figs. 2 and 3 display four regression lines, combining first and second waves (i.e. March–August and September–December) with the R-squares ranging between 0.78 and 0.84. There were highly significant relationships across both the first and second waves between the percentage of confirmed cases on the one hand, and weekly totals of hospitalized patients, ICU patients and deceased on the other.

The next section discusses the results of the model estimations reported, and implications, as well as key lessons learnt from the COVID-19-related healthcare data across the three time-periods.

## Discussion

The reported COVID-19-related healthcare statistics and diagrams based on Sweden, demonstrate explanatory and predictive power between the percentage of confirmed cases on the one hand, and the weekly totals of hospitalized patients, ICU patients and deceased on the other. We contend that the logic of model estimation and results reported offer relevance to other countries as well.

Early in the pandemic, the model estimations and results indicated that the effects of COVID-19 were going to cause large numbers of hospitalized patients, ICU patients and deceased^1^. The first wave of COVID-19 healthcare data provided an opportunity to gather experience and insights in order to handle a second wave with pre-determined, preventive and restrictive countermeasures.

The first wave opened-up a window of model estimations for assessing the predictability of COVID-19- related morbidity and mortality, not only in Sweden, but in other countries as well. Model estimations after the first wave could have established a satisfactory predictability of hospitalized patients, ICU patients and deceased. This could thus have been used by governments and agencies of public health in their pandemic strategies and epidemic plans of action to impose enhanced countermeasures to face a second wave in advance. Proactive protocols could have been established about what, when, where, who and how to react and act by imposing pre-determined, preventive and restrictive countermeasures in the case of a second wave.

With the facts on hand derived from the COVID-19-related healthcare data from the first wave, many governments and agencies of public health appear to have disregarded the predictability of COVID-19-related healthcare data on morbidity and mortality. Epidemic scenarios provided guidance, while the epidemic curves and related reproduction ratios (R_0_) captured most of the attention and countermeasures appears to have been adapted to them across countries. The drawback of the epidemic curve and reproduction ratios (R_0_) is that they are after-the-fact points of reference for determining measures to handle the pandemic and epidemic, both in the present as well as in the future. Subsequently, these measures have turned out to be reactive rather than proactive.

**Figure 4:**
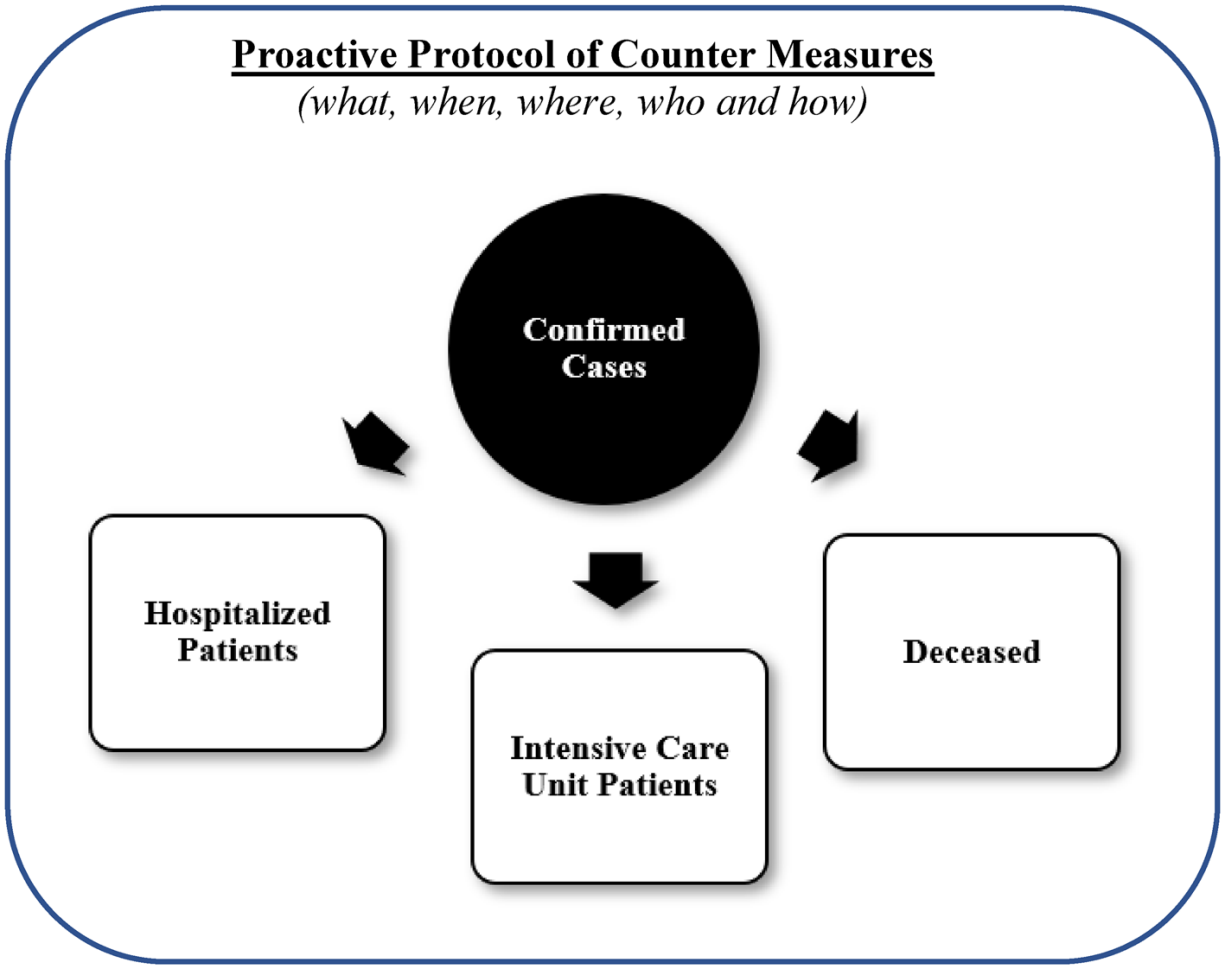
A model estimation and proactive protocol of counter measures.

We therefore contend that pre-determined fixed numbers or thresholds of confirmed COVID-19 cases (≥ 0) could have been applied as reference points to impose pre-determined, preventive and restrictive measures in many countries. The reported logic of model estimation of COVID-19-related morbidity and mortality in Fig. 4 shift the focus from the epidemic curve being a key point of reference to react and act, to instead determining in advance countermeasures to be imposed, based on pre-specified numbers or percentages of confirmed cases (e.g. the higher, the more preventive measures and stricter restrictions imposed, and vice versa).

In line with this, there are countries (such as Australia, China, New Zealand and Taiwan) that appear to have applied a zero-tolerance protocol of confirmed cases to react and act with at least to some extent pre-determined, preventive and restrictive countermeasures, such as implementing immediate lockdowns^7–12^ or travel restrictions^13^, and others (e.g. Finland, Norway, South Korea and Vietnam) with minor-tolerance protocols^14–16^, while other countries (e.g. Brazil, Sweden, UK and USA) applied major-tolerance protocols to react and act, based on a post hoc reliance on epidemic scenarios, epidemic curves and reproduction ratios^17^.

Furthermore, it appears that several Asian countries have reutilized and applied pre-established protocols^11,16^ to face different pandemic and epdidemic scenarios based on previous experiences (e.g. SARS-CoV-1 in 2003 and MERS in 2012). It appears that the zero-tolerance protocols implicitly applied the logic of the reported model estimation, taking into account the predictable consequences that the number or percentage of confirmed cases would have on morbidity and mortality in the society. In fact, we contend that the logic of model estimation can be used as a generic decision tool along with generated predictors to determine a proactive protocol of what, when, where, who and how countermeasures should be imposed, that is, pre hoc rather than ad hoc or post hoc.

A proactive protocol of pre-determined, preventive and restrictive counter measures requires the consideration of a country’s contextual and societal circumstances, such as legislative and judicial boundaries, economic pros and cons, social consequences and cultural acceptability. A non-exhaustive list of countermeasures in a proactive protocol may rest on the questions of what, when, where, who and how to react and act, imposing pre-determined, preventive and restrictive countermeasures.

The initial question is ‘what’ countermeasures are available in the tool box to elaborate a proactive protocol for formulating a pandemic strategy and creating an epidemic plan of action. One of the strongest countermeasures that was imposed during the COVID-19 pandemic in many countries worldwide is the lockdown (i.e., the closure of society) including home confinement of entire populations^8,9,13^.

Societal lockdown in a country is economically and socially, undoubtedly a highly intrusive and extremely restrictive countermeasure. A crucial criterion of a lockdown protocol is therefore to establish ‘when’ it should be imposed and for how long. Another criterion of a lockdown protocol is to establish ‘where’ it should be imposed, such as a specific residential area, city, region or the entire country. An additional criterion of a lockdown protocol is to establish who’ is going to be affected (e.g. activities in crucial sectors of society may be excluded, such as healthcare, police force, fire brigade, energy and food supply and vital transport). Ultimately, a criterion of a lockdown protocol is to establish ‘how’ pre-determined, preventive and restrictive countermeasures should be implemented, so that they become effective (e.g. reducing the spread of infection, morbidity and mortality) and efficient (e.g. optimizing economic costs and social impact).

Consequently, the reported logic of model estimation with a proactive protocol of countermeasures illustrated in Fig. 4 appears not to have been applied, nor have the outcomes of morbidity and mortality been estimated in many countries^17^, though a few may have applied it implicitly or explicitly^11^.

Proactive protocols of pre-determined, preventive and restrictive countermeasures appear not to have been in place across many countries^17^. Rather, they seem to have been post hoc countermeasures, as opposed to pre hoc ones, taking into account the outcome of available healthcare data of model estimation after the first wave. This data could have been used to establish proactive protocols of what, when, where, who and how to react and act, in order to impose countermeasures to meet a second wave. It appears that some countries in Asia and Oceania (e.g. Australia, China, New Zealand and Taiwan) have had proactive protocols of pre-determined, preventive and restrictive measures in place^8,10^.

The Swedish data reported in this study, with high R-squares between the percentage of confirmed cases on the one hand and morbidity as well as mortality on the other, in the first wave appear to have been neglected. In fact, the R-squares became even higher in the second wave. The disregard of a proactive protocol of pre-determined, preventive and restrictive countermeasures guided by pre-fixed numbers or a percentage of confirmed cases, may have contributed to the second wave’s higher rates of morbidity and mortality in Sweden and other countries as well, because of not reacting and acting sufficiently and in advance or at least reasonably quickly. Consequently, lessons that could and should have been learned from the first wave appear to have been disregarded as a means of effectively facing the second wave.

The predictability of COVID-19-related healthcare data outlined on the basis of the logic of model estimation regarding morbidity and mortality in Fig. 4, therefore provides an opportunity and foundation for elaborating proactive protocols of pre-determined, preventive and restrictive countermeasures to formulate a pandemic strategy and create an epidemic plan of action. The reported model estimations show that, if data is available regarding the percentage of confirmed cases, it enables in advance, the estimation of expected numbers of morbidity and mortality in the society.

These are model estimations that can forsee human suffering and death in the population. They can also be used to determine economic costs and social impact, as well as the benefits of countermeasures. Therefore, this study not only provides empirical evidence that COVID-19-related healthcare data is interconnected, but also interrelated, having significant explanatory and predictive abilitites to react and act pre hoc, rather than ad hoc or post hoc. As such, the logic of model estimation offers guidance for elaborating proactive protocols of pre-determined, preventive and restrictive countermeasures in order to formulate a pandemic strategy and create an epidemic plan of action, all of which may be estimated based on different scenarios in connection with the estimated severity of a contagious and virulent virus in society.

We therefore contend that the logic of model estimation is applicable in different settings. Naturally, the outcome of model estimations will vary between countries, but by having the COVID-19-related healthcare data available in a society, the consequences of morbidity and mortality could have been estimated satisfactorily. It could thus have enabled governments and public health agencies to assess in advance the impact on health, social and economic costs. It could have shed light on the impact that the percentage of confirmed cases can have, such as: (1) health issues in the population; and (2) healthcare resources, such as professionals, equipment, medicines and medical treatments. It could also have shed light on the indirect impact on: (1) sick leave in the labour market; (2) school absenteeism; and (3) care and nursing at old age homes; (4) travel restrictions; and (5) closures of non-vital commercial and hospitality businesses.

We believe it is likely that the introduced logic of model estimation may have been used to some extent in the other Nordic countries, as well as several European ones, based on the preventive and restrictive countermeasures. COVID-19 has affected many countries in a similar way sooner or later in relation to the elevated numbers of morbidity and mortality in the populations in question. Preventive and restrictive countermeasures (i.e. what, when, where, who and how) differ between countries and may explain why some countries have succeeded better than others, and vice versa.

It is therefore likely that the explanatory and predictive ability of model estimations reported in this study will resemble those in other countries, taking to account the preventive and restrictive countermeasures. Consequently, COVID-19-related healthcare data appears to be strongly interrelated. Evidently, there are other circumstances that will also effect the COVID-19-related healthcare data, such as the economic, social and cultural circumstances in each country. Nevertheless, the reported logic of model estimation and proposed proactive protocol of counter measures, provides guidance in other settings, although differences will certainly occur.

We therefore contend that the logic of model estimation for COVID-19- related morbidity and mortality to some extent possesses generality to other pandemic and epidemic situations. For example, it may have been applicable to other virulent viruses (e.g. SARS-CoV-1 and MERS), although the model estimations and related predictors had to be re-visited and subsequently re-calculated. The reported logic of model estimation can therefore be relevant to viruses causing virulent seasonal flues that may strike countries on occasion (i.e. not necessarily annually), though the severity of morbidity and rates of mortality is not comparable to the COVID-19. The model estimations may have similar explanatory and predictive power, although the consequences for morbidity and mortality are less in comparison to COVID-19-related healthcare data.

Nevertheless, it is not the last time a pandemic will strike the world, and the question is therefore when the current one will end and the next one will emerge. Though several vaccines have been developed and approved, and vaccinations are taking place on a large scale, the end of the pandemic is regrettably not around the corner. It may still endure longer than expected as mutated viruses (e.g. the British one labelled ALFA and the Indian one labelled DELTA) are emerging continuously around the world^18^ and will continue to emerge down the line. It is therefore relevant to consider at early stages, the explanatory and predictive patterns revealed, based on the logic of model estimation regarding COVID-19-related healthcare data reported in this study.

The model estimations contribute a counterweight to the epidemic curve by turning the ballgame around to pre-fixed numbers or thresholds of confirmed cases, thus enabling reacting and acting with pre-determined, preventive and restrictive countermeasures, which can be updated on a weekly basis. Another contribution is the explanatory and predictive ability of COVID-19-related healthcare data, such as, if there is a spread of a contagious and virulent virus in the society, the morbidity and mortality can be estimated in advance at an early stage. A third one is that the logic of model estimation assists in the implementation of a pandemic strategy and epdidemic plan of actions containing a proactive protocol regarding what, when, where, who and how pre-determined, preventive and restrictive countermeasures should be in place when a virulent virus or pandemic strikes again. A fourth one is that of shedding light on the sequence of reactions and actions needed in the early identification of confirmed cases and the subsequent chain of transmission in the society. If the disease-transmission chain is interrupted, or at least restricted, the reported model estimations indicate that the morbidity and mortality can indeed be controlled.

In conclusion, we contend that relevant and valuable lessons for the future can be learnt by taking into account the reported model estimations. One lesson is that COVID-19-related morbidity and mortality in a population are predictable. Another lesson is the need to have a proactive protocol of countermeasures in place.

## Methodology

This section contextualizes the Swedish pandemic and epidemic setting in relation to the other Nordic countries, and also explains the methodological procedures applied in this study.

The Swedish approach to handling the pandemic strategy and epidemic plan of action differs from neighboring and nearby countries^17^, as well as in the world as a whole, thus providing a distinguishing point of reference to other research contexts.

The planning and implementation of the Swedish pandemic strategy and epidemic plan of action have been characterized by the government and the Agency of Public Health, both of which to a large extent transferred the responsibility for reacting and acting to the population, rather than imposing strict, preventive and restrictive countermeasures in advance, so as to control the spread of the COVID-19^17,18^, as done in neighboring countries, such as Denmark, Finland and Norway, as well as most European countries. In fact, the Government Offices^19^ writes: “…every person in Sweden needs to take responsibility. By everyone taking responsibility theselves, we can keep the level of spread of infection down…”.

The COVID-19-related mortality per capita in Sweden is among the highest in the world^3,4^ , although it is a highly developed economy with a small population and low population density^20^. Furthermore, the population is socially and culturally characerised anyway by the norm of social distancing^21^. Denmark, Finland and Norway are socially and culturally similar to Sweden^22–24^. The Swedish government and the Agency of Public Health have reacted and acted differently to the neighboring countries^17^, and not to the benefit of the country.

The COVID-19-related healthcare statistics between the neighboring countries (Danmark, Finland and Norway) and Sweden reveal major differences^25,26^ , such as: (1) the number of confirmed cases is three times higher per capita in Sweden than in Denmark, Finland and Norway alltogether; (2) the number of performed tests per capita is seven times lower in Sweden than in the neighboring countries together; and (3) mortality per capita is six times lower in the neighboring countries together than in Sweden. Accordingly, the COVID-19-related morbidity per capita (i.e. hospitalized and ICU patients) is therefore also much lower in Denmark, Finland and Norway altogether, compared to Sweden.

The official healthcare-related COVID-19 data was gathered from the Swedish Agency of Public Health^3^, the Swedish National Board of Health and Welfare, and the Swedish Intensive Care Registry—SIR^4,5^. The sets of healthcare data from each entity has been continuously revisited for updates throughout the pandemic. Consequently, the model estimations of COVID-19-related morbidity and mortality in this study are tested according to Swedish official healthcare data.

The model estimations and results are based on the COVID-19-related healthcare data during 42 consecutive weeks, all of which has been divided into three time-periods to separate the first wave of COVID-19 from the second one, and all together as one time-period. The first wave terminated at the end of August (i.e. week number 36) and the beginning of September (i.e. week number 37). The criteria for determining the end of the first wave and the beginning of the second one is based on when the percentage of confirmed cases reached it lowest level^3^ (i.e. 1.1% weeks 36 and 37) since the inception of the pandemic. The subsequent series of statistical analyses and related diagrams are based on three time-periods: (1) from March to August (26 weeks); (2) from September to December (16 weeks); and (3) from March to December (42 weeks).

The model estimations of COVID-19-related morbidity and mortality are based on the weekly totals of healthcare data from the beginning of March 2020 until end of December of the same year^3^. The data analyses start on March 9 (week 11 when the first death of COVID-19 was reported^3^) and ends on December 27 (week 52) when vaccinations were initiated^27^ to enable model estimations to be comparable between the first and second waves, including the time-delays up to 4 weeks of healthcare data in connection with the dependent variables (see Table 1).

It should be noted that the confirmed cases at a given time do not usually lead to hospitalization, ICU or death at the same time, and there is a time-delay. Time-delays between tested Covid-19-related variables are therefore applied in the reported analyses. Nevertheless, a hypothetical situation could refer to a confirmed case on day one, being hospitalized on day two, then admitted to ICU on day three and finally, deceased on day four. In fact, all of this could take place on day one, but alternatively, with a major time-delay.

In reality, there is often a certain time-delay after a case of Covid-19 has been tested and confirmed, and the subsequent effects on the number of hospitalized and ICU patients in the near future, as well as on the time between confirmed cases until patients are deceased. In fact, the time-delay in Sweden between confirmed cases and: (1) patients being hospitalized—on average 6.2 days^4^; (2) patients being submitted to ICU—on average 10.6 days^5^; and (3) until patients are deceased—on average 12.5 days^4^.

Consequently, the averages indicate a range of time-delays. We therefore find it justified to apply time-delays from 0 to 4 weeks in model estimations, to enable comparisons between the weekly percentage of confirmed cases and the number of hospitilized and ICU patients per week, as well as deceased per week. In fact, there are only minor differences between the R-squares reported with time-delays of one and two weeks in Table 1, but the R-squares are lower for weeks zero, three and four. We have therefore determined that primarily one and two weeks are the appropriate range of time-delays on which to focus in the model estimations. One and two week time-delays are therefore prioritized in the results reported. The other time-delays of zero, three and four weeks are also reported to provide the results of all tested model estimations.
